# Chromatin That Guides Dosage Compensation Is Modulated by the siRNA Pathway in *Drosophila melanogaster*

**DOI:** 10.1534/genetics.118.301173

**Published:** 2018-06-19

**Authors:** Nikita Deshpande, Victoria H. Meller

**Affiliations:** Department of Biological Sciences, Wayne State University, Detroit, Michigan 48202

**Keywords:** *Ago2*, dosage compensation, chromatin modification, satellite repeats, 1.688^X^ repeats, 359 bp repeats, *roX1 roX2*, X chromosome recognition

## Abstract

Many heterogametic organisms adjust sex chromosome expression to accommodate differences in gene dosage. This requires selective recruitment of regulatory factors to the modulated chromosome. How these factors are localized to a chromosome with requisite accuracy is poorly understood. *Drosophila melanogaster* males increase expression from their single X chromosome. Identification of this chromosome involves cooperation between different classes of X-identity elements. The chromatin entry sites (CES) recruit a chromatin-modifying complex that spreads into nearby genes and increases expression. In addition, a family of satellite repeats that is enriched on the X chromosome, the 1.688^X^ repeats, promotes recruitment of the complex to nearby genes. The 1.688^X^ repeats and CES are dissimilar, and appear to operate through different mechanisms. Interestingly, the siRNA pathway and siRNA from a 1.688^X^ repeat also promote X recognition. We postulate that siRNA-dependent modification of 1.688^X^ chromatin contributes to recognition of nearby genes. In accord with this, we found enrichment of the siRNA effector Argonaute2 (Ago2) at some 1.688^X^ repeats. Mutations in several proteins that physically interact with Ago2, including the histone methyltransferase Su(var)3-9, enhance the lethality of males with defective X recognition. Su(var)3-9 deposits H3K9me2 on some 1.688^X^ repeats, and this mark is disrupted upon ectopic expression of 1.688^X^ siRNA. Furthermore, integration of 1.688^X^ DNA on an autosome induces local H3K9me2 deposition, but enhances expression of nearby genes in a siRNA-dependent manner. Our findings are consistent with a model in which siRNA-directed modification of 1.688^X^ chromatin contributes to recognition of the male X chromosome for dosage compensation.

## Abstract (precis)

A family of X-linked repetitive elements enhances dosage compensation of nearby genes in male flies. Here, Deshpande and Meller show that chromatin around these repeats is modified in a siRNA-dependent manner. Proteins that interact with the siRNA effector...

MALES of many species carry one X chromosome and a gene-poor Y chromosome. Hemizygosity of the male X chromosome produces a potentially lethal imbalance in the ratio of X to autosomal gene products. This imbalance is corrected by a process known as dosage compensation, a specialized type of gene regulation that modulates expression of an entire chromosome. Different strategies to achieve dosage compensation have evolved independently. In *Drosophila melanogaster*, males increase X-linked gene expression by approximately twofold (Lucchesi *et al.* 2005). This involves the activity of the Male Specific Lethal (MSL) complex. The MSL complex is recruited to active genes on the X chromosome, where it modifies chromatin to increase expression (Lucchesi and Kuroda 2015). The MSL complex contains five proteins, Male-Specific Lethal-1, -2, and -3 (MSL1, -2, -3), Maleless (MLE), and Males absent on the first (MOF) (reviewed in Koya and Meller 2011). Enhanced transcription by the MSL complex is associated with H4K16 acetylation by MOF (Akhtar and Becker 2000; Smith *et al.* 2000). H4K16 acetylation decompacts chromatin, and this may enhance transcriptional elongation of X-linked genes (Shogren-Knaak *et al.* 2006; Larschan *et al.* 2011).

The MSL complex also contains one of two long noncoding *RNA on the X* (*roX1*, *2*) transcripts (Quinn *et al.* 2014). While elimination of any one of the MSL proteins is lethal to males, *roX1* and *roX2* are redundant for compensation. Mutation of both *roX* genes leads to mislocalization of the MSL proteins to ectopic autosomal sites in male larvae (Meller and Rattner 2002; Deng and Meller 2006). X-linked gene expression is reduced in these males, as is survival to adulthood. Both *roX* genes are located on the X chromosome, and both overlap chromatin entry sites (CES)—specialized sites with increased affinity for the MSL complex (Kelley *et al.* 1999; Alekseyenko *et al.* 2008; Straub *et al.* 2008).

Although much is known about the role of MSL complex in dosage compensation, how this complex selectively targets the X chromosome is poorly understood. Recognition and binding to X chromatin is believed to be a two-step process. Initial recruitment of the MSL complex to CES is followed by spreading into nearby transcribed genes (Gelbart and Kuroda 2009). Contained within the CES are motifs called MSL recognition elements (MREs) (Alekseyenko *et al.* 2008; Straub *et al.* 2008). MREs are 21-bp GA-rich motifs that bind chromatin-linked adaptor for MSL protein (CLAMP)—a zinc finger protein that is essential for MSL recruitment (Soruco *et al.* 2013). Spreading into nearby active genes is supported by interaction of MSL3 with the cotranscriptional H3K36me3 mark (Kind and Akhtar 2007; Larschan *et al.* 2007; Sural *et al.* 2008). These mechanisms describe local recruitment of the MSL complex, but fail to explain how the MSL complex specifically targets the X chromosome. H3K36me3 is found on active genes throughout the genome, and MREs are only modestly enriched on the X chromosome. Furthermore, CLAMP binds MREs throughout the genome, but only recruits the MSL complex to X-linked CES (Alekseyenko *et al.* 2008; Soruco *et al.* 2013). We conclude that additional mechanisms must distinguish X and autosomal chromatin.

X-localization is disrupted in *roX1 roX2* males, making them a sensitized genetic background that can be used to identify additional factors contributing to X recognition. Using this strategy, our laboratory demonstrated a role for the siRNA pathway in recognition of the X chromosome (Menon and Meller 2012; Menon *et al.* 2014). A likely source of siRNA is a family of repeats that is near exclusive to the X chromosome. These are the AT-rich, 359-bp 1.688^X^ satellite repeats, a clade of which is found in short, tandem arrays throughout X euchromatin (Hsieh and Brutlag 1979; Waring and Pollack 1987; DiBartolomeis *et al.* 1992; Gallach 2014). Specific clusters are denoted by a superscript indicating cytological position. In support of the idea that 1.688^X^ repeats assist X recognition, ectopic production of siRNA from one repeat partially rescues *roX1 roX2* males (Menon *et al.* 2014). 1.688^X^ repeats are often close to or within genes, leading to the idea that they function as “tuning knobs” for gene regulation (Kuhn *et al.* 2012). In accord with these ideas, autosomal insertions of 1.688^X^ DNA enable recruitment of functional dosage compensation to nearby autosomal genes (Joshi and Meller 2017).

The 1.688^X^ repeats share no sequence identity with the CES, and appear to act in a genetically distinct manner (Joshi and Meller 2017). The question of how 1.688^X^ DNA promotes compensation of nearby genes is thus of great interest. We pursued the idea that siRNA-directed modifications of chromatin at 1.688^X^ repeats link the repeats and the siRNA pathway to X recognition. Reduction of the siRNA-binding effector protein Argonaute2 (Ago2) enhances the lethality of partial loss of function *roX1 roX2* mutations, and further reduces X-localization of MSL proteins (Menon and Meller 2012). We hypothesized that an Ago2-containing complex might localize to and modify 1.688^X^ chromatin in otherwise wild-type flies. In accord with this idea, we find that Ago2 is enriched at 1.688^X^ repeats. Proteins interacting with Ago2 may also play a role in dosage compensation. To address this, we tested high confidence Ago2-binding proteins for genetic interactions with *roX1 roX2*, and found that mutations in several of these genes further reduced the survival of *roX1 roX2* males. Of particular interest is the H3K9 methyltransferase *Su*(*var*)*3-9*, which is responsible for enrichment of H3K9me2 at a subset of 1.688^X^ repeats. H3K9me2 enrichment is disrupted upon ectopic expression of 1.688^X^ siRNA. Chromatin flanking an autosomal insertion of 1.688^X^ DNA is enriched for H3K9me2, and enrichment is enhanced by ectopic expression of 1.688^X^ siRNA. In contrast to the repressive nature of H3K9me2, we find that expression of autosomal genes close to the 1.688^X^ transgene is increased in male larvae, and further elevated by additional 1.688^X^ siRNA. These findings support the idea that X recognition and transcriptional upregulation by dosage compensation are distinct processes, and suggest that siRNA-dependent modification of chromatin in or near 1.688^X^ repeats contributes to X recognition in wild type flies. We propose that epigenetic modifications link the siRNA pathway, 1.688^X^ repeats on the X chromosome and X recognition.

## Materials and Methods

### Fly culture and genetics

Mutations *Dcr1^Q1147X^* (BDSC #32066), *Rm62^01086^* (BDSC #11520), *Fmr1^Δ113m^* (BDSC #67403), *Su*(*var*)*3-9^1^* (BDSC #6209), S*u*(*var*)*3-9^2^* (BDSC #6210), *smg^1^* (BDSC #5930), *Taf11^1^* (BDSC #65410), *Taf11^5^* (BDSC #65409), *p53^5A-1-4^* (BDSC #6815), *p53^11-1B-1^* (BDSC #6816), *foxo^Δ94^* (BDSC #42220), *PIG-S^e00272^* (BDSC #17833), *bel^L4740^* (BDSC #10222), *bel^6^* (BDSC #4024), *barr^L305^* (BDSC #4402), *SmD1^EY01516^* (BDSC #15514), v*ig^C274^* (BDSC #16323), *Ago1^k08121^* (BDSC #10772), *aub^QC42^* (BDSC #4968), *piwi^06843^* (BDSC #12225), *Su(var)2-10^2^* (BDSC #6235), *egg^MB00702^* (BDSC #22876), *G9a^MB11975^* (BDSC #29933), P{EPgy2}*^09821^* (BDSC #16954), P{EPgy2}*^15840^* (BDSC #21163), and *FLAG.HA.Ago2* (BDSC #33242)were obtained from the Bloomington *Drosophila* Stock Center. *Ago2^414^* (Kyoto #109027) was obtained from the Kyoto Stock Center. *Su*(*var*)*3-7^14^* was a gift from Dr. P. Spierer (Seum *et al.* 2002). *ocm^166^* was a gift from Dr. R. Kelley. *ΔDsRedΔupSET* (*upSET* in Figure 2) was a gift from Dr. M. Kuroda (McElroy *et al.* 2017). To minimize genetic background effects all mutations were outcrossed for five generations using a nearby marked *P*-element (unmarked mutations) or the laboratory reference *yw* strain (mutations marked with *w^+^* or *y^+^*). Stocks were constructed with outcrossed, rebalanced chromosomes, and a reference Y-chromosome (Menon and Meller 2009). All mutations were confirmed by phenotype or PCR. Mating schemes to determine the effect of Ago2-interactors on dosage compensation are presented in Supplemental Material, Figure S1. Each test scored ∼1000 flies and was performed in triplicate. To express 1.688^3F^ siRNA in a *Su*(*var*)*3-9^−/−^* mutant background, we generated [*hp1.688^3F^*] [*Sqh*-Gal4]*/In*(2LR)*Gla wg^Gla-1^*; *Su*(*var*)*3-9^1^/ TM3TbSb* flies and selected non-*Tb* third instar males for ChIP. The [*Sqh*-Gal4] insertion was a gift of Dr. S. Todi. The [*hp1.688^3F^*] transgene contains part of the 1.688^3F^ repeat cluster coned in inverted orientation in pWIZ (Sik Lee and Carthew 2003). Although siRNA accumulates to abnormally high levels in larvae expressing [*hp1.688^3F^*], the siRNAs produced appear similar to those isolated from wild type embryos (Menon *et al.* 2014)

### Tissue collection and chromatin preparation

Embryo collection and chromatin preparation was as previously described (Koya and Meller 2015). Briefly, 0.5 g of 0–12 hr embryos were collected on molasses plates with yeast. Embryos were dechorionated for 2.5 min in bleach, cross-linked in 50 mM HEPES, 1 mM EDTA, 0.5 mM EGTA, 100 mM NaCl, 1% formaldehyde with heptane for 20 min. Cross-linking was quenched with 125 mM glycine, 0.01% Triton X-100, 1× PBS for 30 min. Embryos were washed with 10 mM HEPES, 200 mM NaCl, 1 mM EDTA, 0.5 mM EGTA and 0.01% Triton X-100, and suspended in 2.5 ml of 10 mM HEPES, 1 mM EDTA, 0.5 mM EGTA, 0.1% Na-deoxycholate and 0.02% Na-azide for sonication. Sonication, performed on ice at 35% amplitude, 30 sec on, 59 sec off for a total time 15 min using a Fischer Scientific Model FB505 sonicator, produced 300–600 bp fragments. Chromatin was clarified by centrifuging at 13,000 rpm for 15 min, diluted 1:1 with 2× RIPA buffer [2% Triton X-100, 0.2% Na-deoxycholate, 0.2% SDS, 280 mM NaCl, 20 mM Tris-HCl pH 8.0, 2 mM EDTA, 0.02% Na-azide, 2 mM DMSF with complete protease inhibitor (Roche)]. Chromatin solution (5.5 ml) was preabsorbed by incubation at 4° for 30 min with 55 µl of blocked Pierce Protein A agarose beads (Catalog #20333) and aliquots stored at −80°.

For larval chromatin, a modified protocol from Kuzu *et al.* (2016) was used. 150 larvae were frozen in liquid N_2_ and ground in a chilled mortar. The powder was transferred to a cooled 15 ml Dounce and homogenized with a loose pestle (10 strokes) and a tight pestle (15 strokes) in 10 ml PBS with protease inhibitor. Homogenate was made to 40 ml with PBS, cross-linked with 1% formaldehyde for 20 min, and quenched with 125 mM glycine for 30 min. Cross-linked material was pelleted, washed once with wash buffer A (10 mM Hepes pH 7.6, 10 mM EDTA, 0.5 mM EGTA, 0.25% Triton X-100, protease inhibitor and 0.2 mM PMSF), once with wash buffer B (10 mM Hepes pH 7.6, 100 mM NaCl, 1 mM EDTA, 0.5 mM EGTA, 1% Triton X-100, protease inhibitor and 0.2 mM PMSF), and three times with TE wash buffer (10 mM Tris pH 8.0, 1 mM EDTA, 0.01% SDS, protease inhibitor, and 0.2 mM PMSF). The pellet was resuspended in 2 ml pre-RIPA buffer (0.1% SDS, 10 mM Tris-HCl, 1 mM EDTA, protease inhibitor, and 0.2 mM PMSF). Sonication was performed at settings described above for 2 min. Sonicated samples were diluted with 1% Triton X-100, 0.1% Na-deoxycholate, and 140 mM NaCl, centrifuged at 1500 × *g* to clarify, aliquoted, and stored at −80°.

### Chromatin immunoprecipitation

Chromatin (75 μg) was incubated overnight at 4° with 4 µl anti-H3K9me2 (ab1220; Abcam) or 8 µl anti-H3K9me3 (ab8898; Abcam), clarified by centrifugation at 14,000 rpm for 5 min, and supernatants transferred to tubes containing 40 µl blocked Pierce Protein A agarose beads (Catalog #20333) and incubated 4 hr at 4°. Following washing, reverse cross-linking, organic extraction, and precipitation, DNA was suspended in 50 µl distilled water.

### ChIP-qPCR

Duplicate 20 µl reactions consisting of 2 µl DNA, 10 µl Bio-Rad iTaq (#172-5101), and primers were amplified using an Mx3000P Real-Time PCR system (Stratagene). SE was derived from the mean Ct values of biological replicates. Quantitative PCR (qPCR) analysis was previously described (Koya and Meller 2015). Each ChIP pull down was validated. For H3K9me2, primers in an H3K9me2-enriched region of the third chromosome, and *dmn* (DCTN2-p50) served as positive and negative controls. ChIP primers are presented in Table S1. Primer specificity for 1.688^X^ repeats was ensured by anchoring one primer in flanking unique sequence (1.688^1A^, 1.688^3C^, 1.688^4A^, and 1.688^7E^) or by designing primers to unique sequences within repeats and testing with genomic DNA from a strain deleted for the repeat cluster (1.688^3F^ and 1.688^7F^; see Menon *et al.* 2014). Primer efficiencies were determined using MxPro qPCR software. Repeat copy number is normalized by expressing enrichment as percent input.

### Protein isolation from embryos

Embryos (0–12 hr; 50 mg) were homogenized in 250 µl RIPA buffer on ice. Homogenate was passed through a 26 gauge needle 10–12 times to shear DNA. Particulate matter was removed by centrifugation, and supernatant was mixed with an equal volume of 2× SDS sample buffer and boiled for 5 min before separation on a 15% SDS polyacrylamide gel.

### Protein blotting

Polyacrylamide gels were equilibrated in transfer buffer (48 mM Tris, 39 mM glycine, 1.3 mM SDS, 20% methanol) for 20 min. A polyvinylidene difluoride (PVDF) membrane was activated in 100% methanol for 1 min. Filter paper and activated PVDF membranes were saturated in transfer buffer and proteins transferred using a Trans-Blot SD Semi-Dry Transfer Cell (Bio-Rad). The membrane was washed in TBST (10 mM Tris-Cl, 200 mM NaCl, 0.1% Tween 20, pH 7.5), blocked in 5% bovine serum albumin, and probed overnight at 4° using 1:2000 mouse anti-H3K9me2 diluted in blocking solution (ab1220; Abcam) or 1:4000 goat anti-tubulin (E7; Developmental Studies Hydrinoma Bank). After washing with TBST, the membrane was incubated with alkaline-phosphatase-conjugated secondary antibodies (A3562, goat anti-mouse; Sigma, or A4062, rabbit anti-goat; Sigma), washed and developed in 100 mM diethanolamine, 100 mM NaCl, 5 mM MgCl_2_, pH 9.5 containing 33 µg/ml nitroblue tetrazolium (NBT) and 165 µg/ml 5-bromo-4-chloro-3-indolyl phosphate (BCIP). Signals were quantified by ImageJ.

### Quantitative RT-PCR

Total RNA was isolated from 50 third instar male larvae or 100 mg dechorionated embryos using Trizol reagent (Invitrogen) as previously described (Koya and Meller 2015). RNA (1 μg) was reverse-transcribed using random hexamers and ImProm-II reverse transcriptase (Promega). Duplicate reactions were amplified using iTaq Universal SYBR Green Supermix (Bio-Rad) with an Mx3000P Real-Time PCR system (Stratagene). Primers are in Table S1. For determining relative transcript abundance, values were normalized to *dmn*. To calculate fold change, values were normalized to *dmn* and to a reference strain. Expression was calculated using the efficiency corrected comparative quantification method (Pfaffl 2001).

### Data availability

The authors state that all data necessary for confirming the conclusions presented in the manuscript are represented fully within the manuscript. Strains and materials used in this study are available upon request. Supplemental material available at Figshare: https://doi.org/10.25386/genetics.6083165.

## Results

### Ago2 localizes at 1.688^X^ repeats

**Figure 1:**
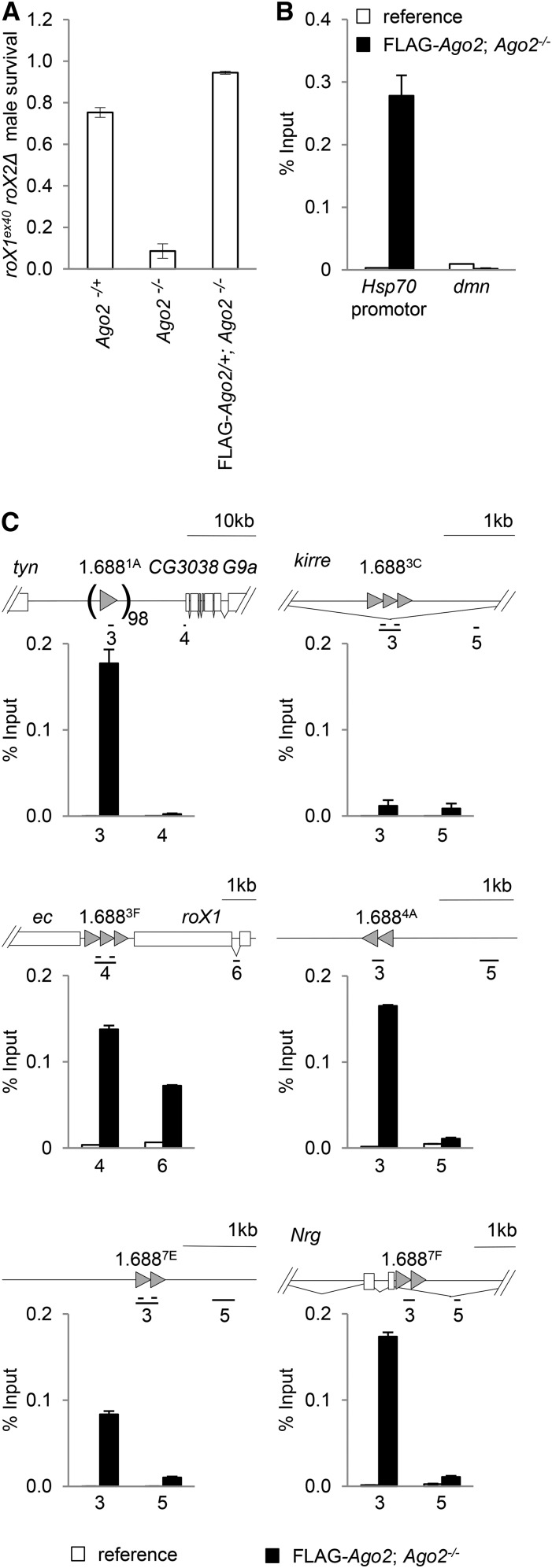
FLAG-Ago2 rescues the Ago2 dosage compensation function and localizes at 1.688^X^ repeats. (A) A FLAG-*Ago2* transgene (right) rescues the synthetic lethality of *roX1^ex40^ roX2Δ*/Y; *Ago2^414/414^* males (center). (B) Chromatin from the laboratory reference strain (white) and *Ago2^414/414^*; FLAG*-Ago2* (black) embryos was precipitated with anti-FLAG antibody. Enrichment normalized to input is shown. The *Hsp70* promoter displays enrichment, but a control region in *dmn* does not. (C) FLAG-Ago2 enrichment is detected at several 1.688^X^ repeats (gray arrowheads). Approximately 100 copies of the 1.688^1A^ repeats are situated between *tyn* and CG3038. The 1.688^3C^ repeats are within a large *kirre* intron (splicing indicated by diagonal lines). Primers, indexed by gene and amplicon number, are presented in Table S1. Amplicon numbers, constant throughout this study, denote regions in selected repeats and flanking regions as indicated on gene models. In (C) only two amplicons per repeat, one including the repeats and in an adjacent region, were analyzed.

We took advantage of the resolution of ChIP and a FLAG-tagged *Ago2* transgene to determine if Ago2 localizes to 1.688^X^ repeats. FLAG-Ago2 was first tested for rescue of the dosage compensation function of Ago2. Males with the partial loss of function *roX1^ex40^roX2Δ* chromosome have high survival, as do *Ago2^−/−^* flies, but synthetic lethality is observed in *roX1^ex40^roX2Δ*/Y; *Ago2^−/−^* males (Menon and Meller 2012). One copy of a FLAG-*Ago2* transgene rescues these males, demonstrating that the FLAG tag does not disrupt the dosage compensation function of Ago2 (Figure 1A). Chromatin from FLAG-*Ago2*; *Ago^−/−^* embryos, and from a reference strain lacking the FLAG-*Ago2* transgene, was immunoprecipitated with anti-FLAG antibodies and enrichment determined by qPCR. FLAG-Ago2 was enriched at the *Hsp70* promoter—a site known to bind Ago2 (Cernilogar *et al.* 2011) (Figure 1B). In contrast, a control region in the *dmn* gene displayed no enrichment. We then examined FLAG-Ago2 enrichment at a panel of six representative 1.688^X^ repeat clusters that differ in location and environment (within, near or far from protein coding genes), transcription level, and sequence (Table 1). Interestingly, five of these show enrichment of FLAG-Ago2 over the repeats, but little or no enrichment in flanking regions (Figure 1C). We conclude that Ago2 localizes at many 1.688^X^ repeats, a finding that is consistent with involvement of Ago2 in siRNA-directed recruitment of chromatin modification at or around these regions.

**Table 1:** Panel of 1.688^X^ repeats used in this study

Repeat		1.688^1A^	1.688^3C^	1.688^3F^	1.688^4A^	1.688^7E^	1.688^7F^
	Cytological position	1A1	3B5	3F3	4A4	7E5	7F3
	Scaffold coordinates on X	204,046–241,257	2,768,669–2,770,136	3,857,647–3,858,186	4,070,631–4,071,316	8,369,270–8,369,783	8,530,749–8,531,260
Copy number	Genomic scaffold	98	3	2.5	2	1.5	1.5
	Lab reference	—	3.5	3.5	2.5	2.5	1.5
Repeat properties	Percent match to 1.688^3F^	89	69	100	95	71	69
	Genomic environment	37 kb of repeats between convergent genes	Intronic	Between convergent genes	Isolated	Isolated	Intronic
Transcription	EST abundance	0	2	9	2	0	26
	qRT PCR*^a^*	0.00026	0.01	0.3	0.1	0.04	1.5
	RNA polII enrichment	—	—	+	—	—	—

Cytological positions and scaffold coordinates were determined from Flybase (Release 6). The copy number at some positions differed between the laboratory reference strain and the genomic scaffold (see File S1). Similarity to 1.688^3F^ was determined by BLAST alignment of a 359 bp monomer. EST abundance is estimated from Flybase assignments. RNA polII enrichment is derived from ChIP-seq of 6–8 hr mesoderm (Monfort *et al.* 2017).

a Quantitative RT-PCR (qRT PCR) is normalized to repeat copy number (see Figure S4).

### An Ago2-interaction network that participates in dosage compensation

**Figure 2:**
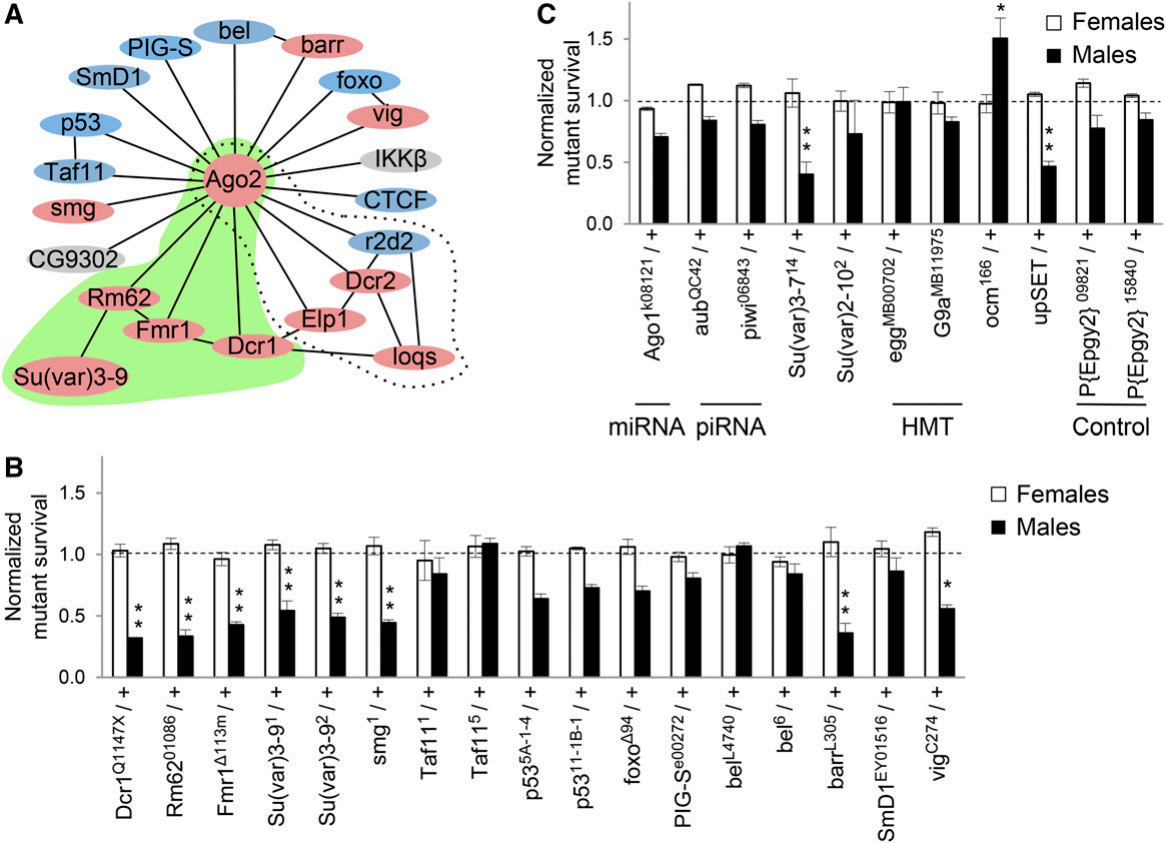
Ago2-interactors participate in dosage compensation. (A) Map of Ago2-interacting proteins. Genes displaying a genetic interaction with *roX1^ex33^roX2Δ* are pink, and those for which a significant interaction has not been detected are blue. Genes in gray are untested. A previously reported siRNA-producing subnetwork is denoted by the dotted line. A putative chromatin-modifying subnetwork identified in the present study is highlighted in green. Well-curated, high probability interactions from BioGRID and esyN are depicted by solid lines. See File S2 for inclusion criteria. (B) Mutations in many Ago2-interacting proteins reduce the recovery of *roX1^ex33^roX2Δ* males (black; *roX1^ex33^roX2Δ/Y*; *mut/+* normalized to *roX1^ex33^roX2Δ/Y*; *+/+*). Females are unaffected (white; *roX1^ex33^roX2Δ/++*; *mut/+* normalized to *roX1^ex33^roX2Δ/++*; *+/+*). (C) Additional controls and genes of interest. The mating strategy to test X-linked G9a is presented in Figure S1B. See *Materials and Methods* for *upSET* description. SEM is represented by error bars. Significance of ≤0.05 (*) and ≤0.001 (**) was determined using the Student’s two sample *t*-test.

Argonaute proteins in the RNA induced transcriptional silencing (RITS) complexes of *Schizosaccharomyces pombe*, and plants recruit chromatin modifiers to nascent transcripts (reviewed in Meller *et al.* 2015). To explore the possibility of Ago2-interacting proteins participating in X chromosome recognition, we screened genes in an Ago2-interaction network for genetic interaction with *roX1 roX2*. A map of high probability Ago2-interactors was created using BioGRID (Stark *et al.* 2006), and esyN (Bean *et al.* 2014) (Figure 2A; see File S2 for inclusion criteria). Members of this network were examined for genetic interactions with the partial loss of function *roX1^ex33^roX2Δ* X chromosome. *roX1^ex33^roX2Δ* males display partial mislocalization of MSL proteins and eclose at 20% of normal levels (Deng *et al.* 2005). Reduction of proteins that participate in X recognition further disrupts X localization and enhances *roX1^ex33^roX2Δ* male lethality (Menon and Meller 2012). Females are fully viable and fertile when the *roX* genes are mutated. *roX1^ex33^roX2Δ* females were mated to males that were heterozygous for a mutation in the gene being tested (Figure S1A). All sons are *roX1^ex33^roX2Δ*/Y, and heterozygous (experimental) or wild type (control) for the gene of interest. A reduction in normalized survival (experimental/control) reveals enhancement of *roX1 roX2* male lethality (Figure 2, B and C). Daughters, which do not dosage compensate and are heterozygous for *roX1^ex33^roX2Δ*, do not display altered survival upon mutation of Ago2-interacting genes. As *G9a* is located on the X chromosome, a modified strategy to test this gene is presented in Figure S1B.

Normalized survival of *roX1^ex33^roX2Δ* males with mutations in the Ago2-interaction network is presented in Figure 2B. Genes displaying significant interactions are noted by pink symbols, and those showing no interaction are blue in Figure 2A. We confirmed a previously identified siRNA-processing subnetwork containing *Dcr2*, *Elp1*, and *loqs* (Figure 2A, dotted line; Menon and Meller 2012). The present study identified several additional Ago2-interactors, including a potential chromatin-modifying subnetwork containing *Dcr1*, *Fmr1*, *Rm62*, and the histone methyltransferase *Su*(*var*)*3-9* (green, Figure 2A). Su(var)3-9 deposits H3K9me2 and acts with Rm62 to resilence active chromatin (Boeke *et al.* 2011).

Additional chromatin modifiers and genes in other small RNA pathways were also tested (Figure 2C). A previous study found no interaction between *roX1^ex33^roX2Δ* and the piRNA pathway genes *aub* and *piwi*, or the miRNA pathway gene *Ago1*, a finding replicated here (Menon and Meller 2012). Since our findings point toward involvement of chromatin modifiers, we tested the chromatin regulatory factor *Su*(*var*)*2-10* and two additional H3K9 methyltransferases, *eggless* (*egg*), and *G9a* (Figure 2C). None of these modified *roX1^ex33^roX2Δ* survival. Mutations in *Su*(*var*)*3-7*, important for heterochromatin formation, and *upSET*, which maintains heterochromatin and H3K9me2 levels, enhance *roX1^ex33^roX2Δ* male lethality (Spierer *et al.* 2008; McElroy *et al.* 2017). *Over compensating males* (*ocm*) has an unusual dosage compensation phenotype as mutations in *ocm* rescue males with insufficient MSL activity, suggesting that it might act as a governor of activation (Lim and Kelley 2013). Interestingly, mutation of *ocm* significantly increased the survival of *roX1^ex33^roX2Δ* males, supporting the idea that *ocm* normally restrains activation. The P{EPgy2}*^09821^* and P{EPgy2}^*15840*^ strains, used to outcross *Su*(*var*)*3-9* and *barr* mutants, display no interaction and serve as controls for genetic background. Taken together, these findings suggest that several genes that deposit H3K9me2, maintain this mark or participate in heterochromatin formation also contribute to X chromosome dosage compensation. At first glance these observations appear to be at odds with X chromosome hypertranscription—the ultimate consequence of X chromosome recognition.

### Ectopically expressed 1.688^3F^ siRNA disrupts H3K9me2 patterns

**Figure 3:**
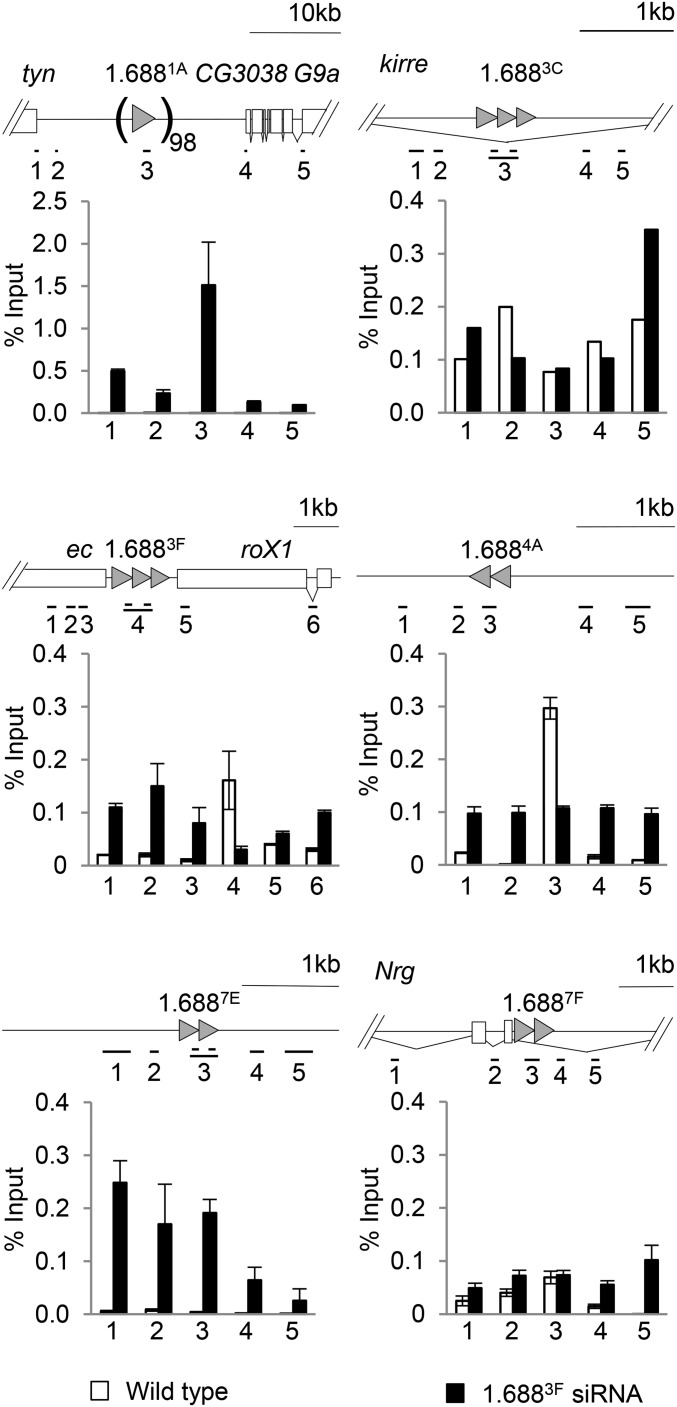
Elevated 1.688^3F^ siRNA disrupts H3K9me2 enrichment around 1.688^X^ repeats. Chromatin from wild type embryos (white) and embryos ectopically producing 1.688^3F^ siRNA (black) was immunoprecipitated with antibody to H3K9me2. Enrichment over input was determined by quantitative PCR (qPCR). The SE of two biological replicates is shown. Amplicons correspond to numbered positions on the gene models above. Primers are presented in Table S1.

Previous studies found that ectopically produced 1.688^3F^ siRNA partially rescues *roX1 roX2* males and increases X localization of the MSL complex (Menon *et al.* 2014). The mechanism by which siRNA promotes X recognition is unknown. The discovery that insertion of 1.688^X^ DNA on an autosome enables functional compensation of nearby genes, and the enhancement of this effect by ectopic 1.688^3F^ siRNA, suggests siRNA action through cognate genomic regions (Joshi and Meller 2017). In accord with this idea, an autosomal *roX1* transgene also enables compensation of nearby genes, but is unaffected by 1.688^3F^ siRNA. To test the idea that 1.688^3F^ siRNA directs epigenetic modification of 1.688^X^ chromatin, we used ChIP to analyze chromatin in and around 1.688^X^ repeats on the X chromosome. ChIP-qPCR detected H3K9me2 enrichment in four out of six repeats (white bars, Figure 3). As H3K9me2 enrichment was not uniform, we considered additional factors that might determine this mark, and noted that only repeats showing evidence of transcription were enriched for H3K9me2, consistent with the idea of Ago2-dependent recruitment to nascent transcripts (Figure S4) (Verdel *et al.* 2004). Upon ectopic expression of 1.688^3F^ siRNA a dramatic disruption of H3K9me2 was observed in and around 1.688^X^ repeats (black bars, Figure 3). For example, 1.688^3F^ and 1.688^4A^ display peaks of H3K9me2 in wild-type flies, but this mark was reduced over the repeats and increased in surrounding regions by elevated 1.688^3F^ siRNA. The reduction in H3K9me2 over repeats themselves was unexpected and could represent repositioning of nucleosomes or blocking by another protein. Untranscribed repeat clusters at 1.688^1A^ and 1.688^7E^ show no H3K9me2 enrichment in wild type flies, but gained H3K9me2 upon expression of 1.688^3F^ siRNA. In contrast, no enrichment of H3K9me3 in or near 1.688^X^ repeats was detected in wild-type or 1.688^3F^ siRNA-expressing embryos (Figure S2). We conclude that some 1.688^X^ repeats are enriched for H3K9me2, and that cognate siRNA broadly disrupts this mark within and several kb adjacent to 1.688^X^ DNA.

### Su(var)3-9 deposits H3K9me2 at 1.688^X^ repeats

**Figure 4:**
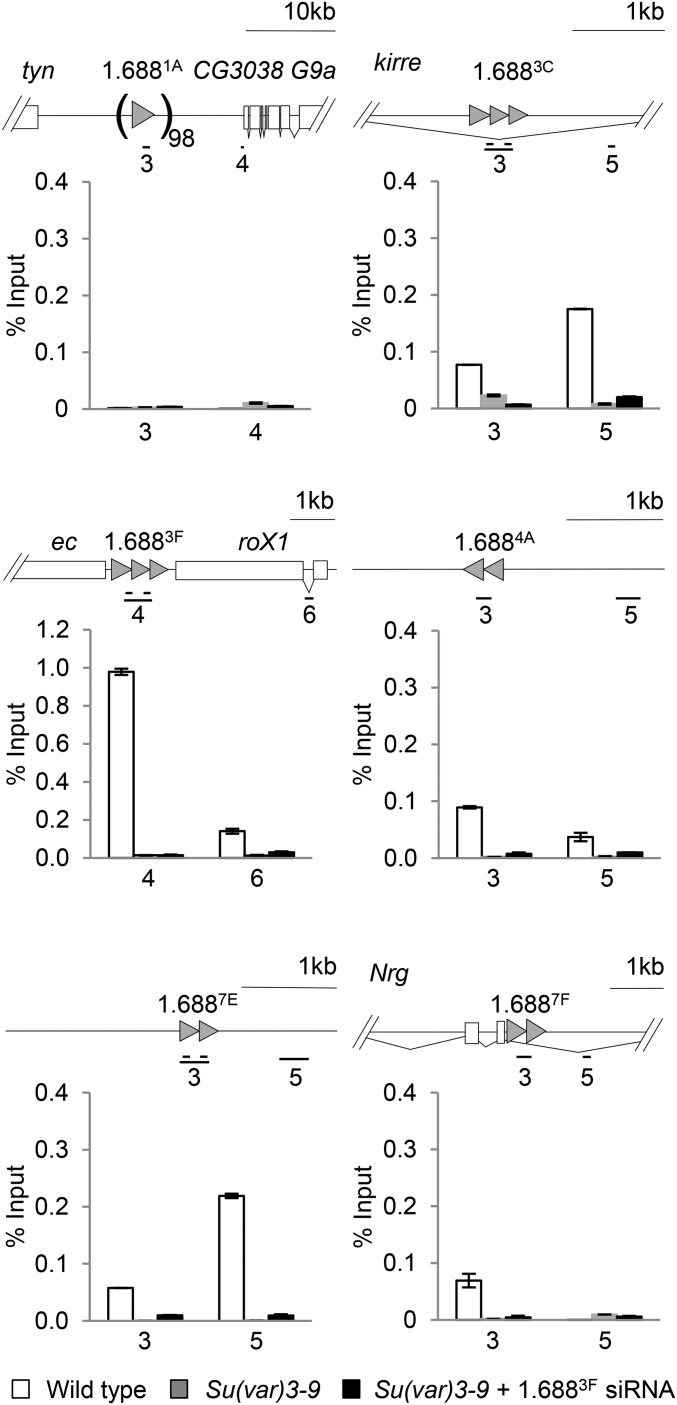
Su(var)3-9 deposits H3K9me2 at 1.688^X^ repeats. Chromatin from wild type male larvae (white), *Su*(*var*)*3-9^1^*/*Su*(*var*)*3-9^1^* male larvae (gray), and *Su*(*var*)*3-9^1^/Su*(*var*)*3-9^1^* males ectopically expressing 1.688^3F^ siRNA (black) was immunoprecipitated with antibody to H3K9me2. Enrichment normalized to input is shown. SE is derived from two biological replicates. See *Materials and Methods* for full genotypes and larval selection strategy.

The identification of Su(var)3-9 as an indirect binding partner of Ago2, observation of a genetic interaction between *roX1 roX2* and *Su*(*var*)*3-9* and enrichment of H3K9me2 on some 1.688^X^ repeats suggested that Su(var)3-9 could be modifying 1.688^X^ repeats. *D. melanogaster* has three histone H3K9 methyltransferases, *Su*(*var*)*3-9*, *eggless*, and *G9a*, but only *Su*(*var*)*3-9* mutations enhance the male lethality of *roX1 roX2* (Figure 2; (Swaminathan *et al.* 2012)). To determine if Su(var)3-9 is responsible for H3K9me2 at 1.688^X^ chromatin, we generated strains carrying *Su*(*var*)*3-9* over a marked balancer, enabling selection of homozygous *Su*(*var*)*3-9* mutant larvae. H3K9me2 enrichment is virtually eliminated over 1.688^X^ repeats in *Su*(*var*)*3-9*^−/−^ mutants (Figure 4, gray) and remains low in *Su*(*var*)*3-9^−/−^* larvae that express 1.688^3F^ siRNA (Figure 4, black). This reveals that Su(var)3-9 deposits H3K9me2 at 1.688^X^ chromatin in wild type flies, and eliminates the possibility that a different methyltransferase is recruited to these regions following ectopic expression of 1.688^3F^ siRNA. Disruption of H3K9me2 upon expression of 1.688^3F^ siRNA thus reflects changes in the localization or activity of Su(var)3-9.

To determine how far from 1.688^X^ repeats the H3K9me2 disruption extends, regions 10–26 kb from repeats were examined. In each case, increased H3K9me2 was observed in embryos with ectopic 1.688^3F^ siRNA expression (Figure S3A). This suggested the possibility of a global change in H3K9me2 levels. To address this we probed protein blots from wild type and 1.688^3F^ siRNA-expressing embryos to determine global levels of this modification, but found no evidence of a change in H3K9me2 (Figure S3B). As most H3K9me2 is found in heterochromatic regions that comprise >30% of the fly genome, the changes we detected in euchromatin may represent a negligible portion of the nuclear pool.

H3K9me2 is generally thought to be repressive, but compensation in flies occurs by increased expression of X-linked genes. To determine if changes in H3K9me2 enrichment correlate with changes in transcription, regions flanking 1.688^X^ repeats were examined in wild-type and 1.688^3F^ siRNA-expressing embryos. Consistent with H3K9me2 having a repressive effect, 1.688^3F^ siRNA decreases accumulation of mRNA, as well as noncoding intragenic and intronic regions, with elevated H3K9me2 (Figure S4). The apparent increase in 1.688^3F^ expression presumably originates from the transgene used to produce ectopic 1.688^3F^ siRNA. We detected dramatic reductions in messages immediately adjacent to 1.688^1A^ (*tyn*, *G9a*) and 1.688^3F^ (*ec*, *roX1*). In spite of a 90% reduction in *ec* transcript in embryos expressing 1.688^3F^ siRNA, adults do not display the rough eye *ec* phenotype. It is possible that ectopic 1.688^3F^ siRNA has a more pronounced effect in embryos, whose lack of differentiation may make them particularly susceptible to epigenetic disruption. Mature patterns of chromatin organization are established later in development, and these may be more resilient. To test this, we examined flanking genes in 1.688^3F^ siRNA-expressing third instar male larvae, and found that *tyn*, *G9a*, and *ec* regained wild-type transcript levels, and *roX1* was largely restored (Figure S4). The precise reason for the differences between embryos and larvae are uncertain, but restoration of normal gene expression by the third larval instar is consistent with the lack of phenotype in otherwise wild type flies that ectopically express 1.688^3F^ siRNA (Menon *et al.* 2014).

The finding that animal age influenced response to ectopic siRNA prompted us to determine the time point at which H3K9me2 is established at 1.688^X^ repeats. A possible scenario is that this mark is placed before MSL localization, and acts in some way to guide X recognition. X-Localization of the MSL complex occurs 3 hr after egg laying (AEL) (Rastelli *et al.* 1995; Meller 2003). We measured H3K9me2 enrichment at 1.688^3F^ in embryos before the MSL complex binds to the X (1.5–3 hr), during initial MSL recruitment (3–4 hr), and at 4–6 and 6–12 hr. In contrast to our prediction, H3K9me2 is first detected on 1.688^3F^ between 6 and 12 hr AEL, after X localization of the MSL complex has occurred (Figure S5). We conclude that H3K9me2 at 1.688^X^ repeats is unlikely to guide initial X recognition, but may serve later to facilitate spreading of this mark or enforce the stability of X recognition. As the failure of dosage compensation only kills males at the end of the third instar, a mechanism that acts later in development would have considerable impact.

### H3K9me2 is enriched at regions flanking autosomal 1.688^3F^ transgenes

**Figure 5:**
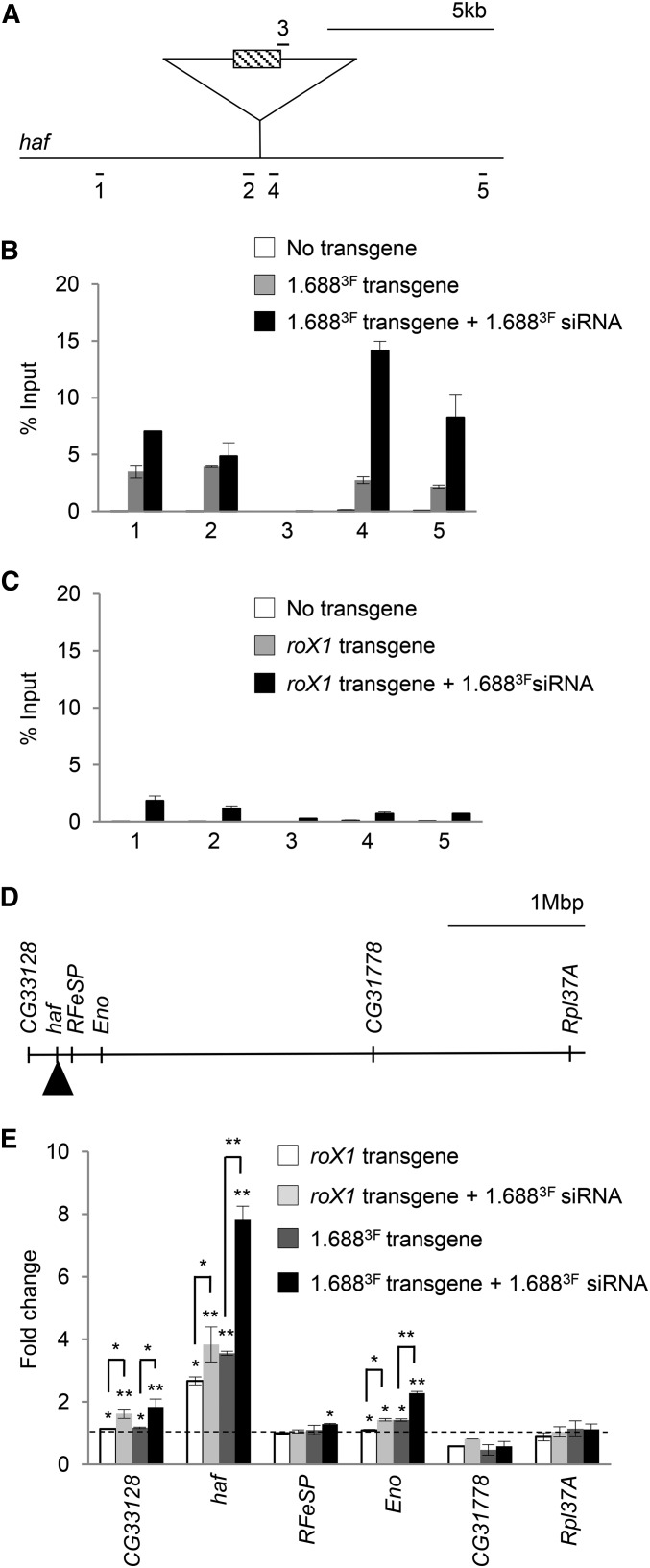
Ectopic 1.688^3F^ siRNA increases H3K9me2 flanking an autosomal 1.688^3F^ DNA insertion and elevates expression of nearby genes. (A) Amplicons flanking the landing site in a large *haf* intron at 22A3 (splicing not shown). (B) H3K9me2 enrichment surrounding the 1.688^3F^ transgene. Chromatin from wild type third instar male larvae (white), larvae with 1.688^3F^ DNA at the landing site (gray), and larvae with 1.688^3F^ DNA at the landing site and ectopic 1.688^3F^ siRNA (black) was immunoprecipitated with antibody to H3K9me2. (C) H3K9me2 enrichment surrounding a *roX1* insertion. Chromatin from wild type male third instar larvae (white), larvae with the *roX1* insertion (gray), and with the *roX1* insertion and ectopic 1.688^3F^ siRNA (black) was immunoprecipitated. Data are from two biological replicates and enrichment is normalized to input. (D) Portion of 2L showing relative location of *CG33128*, *haf*, *RFeSP*, *Eno*, *CG31778*, and *Rpl37A*. (E) Accumulation of transcripts in male larvae carrying *roX1* (white) or 1.688^3F^ insertions (dark gray), and in male larvae that express ectopic 1.688^3F^ siRNA and have *roX1* (light gray) or 1.688^3F^ integrations (black). Expression is normalized to *dmn* and wild type male larvae. SEM is derived from three biological replicates. Significance was determined using Student’s two sample *t*-test, ≤0.05 (*), ≤0.001 (**) significance. Primers are presented in Table S1.

One challenge of studying recruiting elements on the X chromosome is that the redundancy and proximity of these elements complicates interpretation. To overcome this, we tested autosomal integrations of 1.688^3F^ DNA or *roX1* (Figure 5A) (Joshi and Meller 2017). H3K9me2 ChIP was performed on chromatin from third instar male larvae with 1.688^3F^ (Figure 5B) or *roX1* (Figure 5C) on 2L (gray bars), and in the same genotypes with ectopic expression of 1.688^3F^ siRNA (black bars). H3K9me2 within 5 kb of the integration site is not strongly enriched in control males, or in males with a *roX1* transgene, but is striking elevated when 1.688^3F^ DNA is present. Consistent with our observations in embryos, ectopic 1.688^3F^ siRNA further elevated H3K9me2 near the 1.688^3F^ integration. This contrasts with negligible enrichment flanking the *roX1* transgene (Figure 5C). For unknown reasons, enrichment over the integrated 1.688^3F^ DNA was itself undetectable. We conclude that autosomal insertion of 1.688^3F^ DNA makes flanking chromatin subject to siRNA-induced H3K9me2 deposition. Taken together, these studies support the idea that the 1.688^X^ repeats influence patterns of H3K9me2 nearby, but CES-containing *roX1*, with a different class of recruiting element, has little effect.

To determine the influence of 1.688^3F^ and *roX1* on transcription of autosomal genes on 2L, we performed quantitative RT-PCR (qRT PCR) on total RNA from third instar male larvae of the genotypes described above. The 1.688^3F^ and *roX1* integration site is in an intron of *haf*, >17 kb from the closest exon. We also examined *RFeSP*, *CG33128*, *Eno* (30, 89, and 142 kb from the insertion, respectively), and *CG31778* and *Rpl37A*, 2.1 and 3.5 Mb distant (Figure 5D). The presence of 1.688^3F^ or *roX1* integrations alone had no effect on the most distant genes, *CG31778* and *Rpl37A*. A *roX1* integration increased expression of *haf* 2.5-fold, more than expected from full compensation. This may reflect the fact that MSL recruitment to an autosomal *roX1* transgene can overcome local, chromatin-based silencing (Kelley and Kuroda 2003). Addition of 1.688^3F^ siRNA increased *haf* expression slightly, and similarly increased expression of *CG33128* and *Eno* (light gray bars, Figure 5E).

A 1.688^3F^ insertion produced a fourfold increase in *haf*, and a slight increase in *Eno*, 141 kb from the integration site. But, upon expression of 1.688^3F^ siRNA, *haf* expression increased to eightfold wild-type levels, and *CG33128* and *Eno* both increased to twofold wild-type levels, consistent with full compensation. We conclude that an autosomal insertion of 1.688^X^ DNA induces H3K9me2 deposition on flanking chromatin, but also increases expression of genes on 2L in a manner that is consistent with recruitment of the MSL complex. Both H3K9me2 enrichment and increased expression is enhanced by 1.688^3F^ siRNA, suggesting that X identification involves a siRNA-directed mechanism that operates through 1.688^X^ repeats.

## Discussion

Molecularly distinct dosage compensation strategies have arisen independently in different organisms, but a shared feature is the ability to selectively recognize and alter an entire chromosome. How a regulatory system is directed to a single chromosome is poorly understood. The discovery that 1.688^X^ satellite DNA promotes recruitment of dosage compensation to nearby genes supports the idea that these repeats are important for selective recognition of X chromatin (Joshi and Meller 2017). How the 1.688^X^ repeats accomplish this is a question of great interest. Involvement of the siRNA pathway, and siRNA from a 1.688^X^ repeat, in X recognition points to the possibility that siRNA-directed modification of chromatin in and around 1.688^X^ repeats plays a role in dosage compensation in normal males. The findings of the current study support this idea.

Although numerous studies point to small RNA regulation of chromatin in flies, this process is better understood in other organisms (reviewed in Meller *et al.* 2015). Small-RNA-directed heterochromatin formation was discovered in *S. pombe* (reviewed in Moazed 2009). Heterochromatic regions are transcribed during S phase, and transcripts are processed into siRNAs that guide the Ago1-containing RITS complex to complementary, nascent transcripts (Verdel *et al.* 2004). In addition to several other activities, RITS recruits the H3K9 methyltransferase Clr4 (Zhang *et al.* 2008). We propose that a similar process is occurring at 1.688^X^ chromatin in flies. Most 1.688^X^ repeats bind Ago2, and many are transcribed. Several of the 1.688^X^ repeats that we examined are enriched for H3K9me2 deposited by Su(var)3-9—an ortholog of Clr4. Our screen identified genetic interactions between *roX1 roX2* and members of a possible RITS-like complex consisting of Ago2, Rm62 and Su(var)3-9. Finally, H3K9me2 enrichment in, and around, 1.688^X^ repeats is responsive to 1.688^X^ siRNA, and enrichment is blocked by loss of Su(var)3-9. Taken together, these findings suggest that a RITS-like complex normally modifies chromatin at 1.688^X^ repeats.

**Figure 6:**
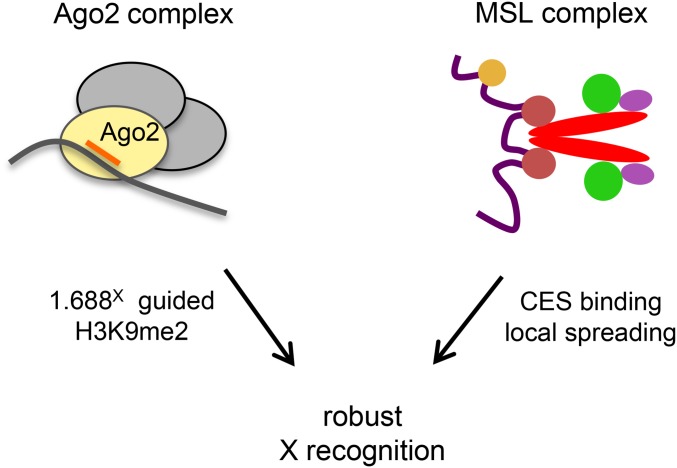
Proposed model of cooperative X recognition. The MSL complex is directly recruited to CES and then spreads into active genes nearby (right). 1.688^X^ siRNA guides an Ago2-containing complex that modifies chromatin around 1.688^X^ DNA, possibly in a transcription-dependent manner (left). We postulate that robust X recognition involves the cooperative action of both pathways.

The idea that repressive H3K9me2 marks participate in a process culminating in a twofold increase in expression is counterintuitive, but X recognition is complex. This process involves CES sites that directly recruit the MSL complex and the 1.688^X^ repeats, acting indirectly to enhance X recognition (Figure 6). It is possible that X recognition uses epigenetic marks, such as H3K9me2, that are distinct from the activating marks deposited within genes by the MSL complex. We propose that robust X recognition results from cooperation between two distinct pathways that guide this process. Interestingly, numerous studies have discovered links between the compensated X chromosome of male flies and repressive marks. For example, the male X is enriched in HP1, a major constituent of heterochromatin that binds H3K9me2 (de Wit *et al.* 2005; Liu *et al.* 2005). The structure of the polytenized male X chromosome is extraordinarily sensitive to altered levels of proteins that participate in heterochromatin formation or silencing, such as *HP1*, *Su*(*var*)*3-7*, and *ISWI*. Mutations of these genes produce a disruption of polytenization that is strikingly specific to the male X (Deuring *et al.* 2000; Spierer *et al.* 2005; Zhang *et al.* 2006). JIL-1, a kinase that enforces boundaries between heterochromatin and euchromatin, is enriched on the X chromosome and thought to participate in compensation (Jin *et al.* 2000; Wang *et al.* 2001; Ebert *et al.* 2004; H. Deng *et al.* 2005). Upon loss of JIL-1, polytenized structure is disrupted and H3K9me2 invades euchromatic chromosome arms, but the X chromosome is most severely affected (Zhang *et al.* 2006). Finally, the MSL proteins themselves have an affinity for heterochromatin. In *roX1 roX2* mutant males, the MSL proteins become mislocalized to ectopic autosomal sites (Meller and Rattner 2002). For reasons that are still unclear, the most prominent of these sites are the heterochromatic fourth chromosome and chromocenter (Deng and Meller 2006; Figueiredo *et al.* 2014). Taken together, these observations suggest that X recognition, or spreading of the MSL complex, could be facilitated by repressive marks. One intriguing possibility is that 1.688^X^ repeats guide deposition of H3K9me2, and this mark, directly or indirectly, assists localization of the MSL complex. Although MSL-mediated compensation initiates at 3 hr AEL, before H3K9me2 enrichment over 1.688^X^ chromatin, male killing due to loss or mislocalization of the MSL complex occurs several days later at the larval/pupal transition. It is possible that ectopic production of 1.688^X^ siRNA drives enrichment of H3K9me2 across the X, supporting X recognition or MSL complex spreading at later developmental stages. This would explain why *roX1 roX2* mutant male larvae, defective for X recognition, display increased X-localization of the MSL proteins and elevated viability upon expression of 1.688^X^ siRNA (Menon *et al.* 2014).

An intriguing aspect of dosage compensation is the evolutionary convergence of mechanisms. For example, long noncoding RNA also plays a central role in X recognition in mammals, where expression of the *X inactive specific transcript* (*Xist*) RNA guides X inactivation (Lee 2009). Furthermore, repetitive LINE-1 elements that are enriched on the mammalian X chromosome are proposed to facilitate X inactivation (Lyon 1998; Bailey *et al.* 2000). Some LINE-1 elements are transcribed during the onset of X inactivation, producing endo-siRNAs that may guide local spreading of heterochromatin into regions that are otherwise prone to escape (Chow *et al.* 2010). These parallels are particularly striking as the outcomes, silencing of an X chromosome in mammalian females and activation of the single X in male flies, appear unrelated. We propose that cooperation between distinct chromatin-modifying systems that rely on long and short noncoding RNAs is one strategy to selectively modulate an entire chromosome.

## References

[bib1] Akhtar A. Becker P. B., 2000 Activation of transcription through histone H4 acetylation by MOF, an acetyltransferase essential for dosage compensation in *Drosophila*. Mol. Cell 5: 367–375. 10.1016/S1097-2765(00)80431-1 10882077

[bib2] Alekseyenko A. A. Peng S. Larschan E. Gorchakov A. A. Lee O. K., 2008 A sequence motif within chromatin entry sites directs MSL establishment on the *Drosophila* X chromosome. Cell 134: 599–609. 10.1016/j.cell.2008.06.033 18724933 PMC2613042

[bib3] Bailey J. A. Carrel L. Chakravarti A. Eichler E. E., 2000 Molecular evidence for a relationship between LINE-1 elements and X chromosome inactivation: the Lyon repeat hypothesis. Proc. Natl. Acad. Sci. USA 97: 6634–6639. 10.1073/pnas.97.12.6634 10841562 PMC18684

[bib4] Bean D. M. Heimbach J. Ficorella L. Micklem G. Oliver S. G., 2014 esyN: network building, sharing and publishing. PLoS One 9: e106035 10.1371/journal.pone.0106035 25181461 PMC4152123

[bib5] Boeke J. Bag I. Ramaiah M. J. Vetter I. Kremmer E., 2011 The RNA helicase Rm62 cooperates with SU(VAR)3–9 to re-silence active transcription in *Drosophila melanogaster*. PLoS One 6: e20761 10.1371/journal.pone.0020761 21674064 PMC3107242

[bib6] Cernilogar F. M. Onorati M. C. Kothe G. O. Burroughs A. M. Parsi K. M., 2011* *Chromatin-associated RNA interference components contribute to transcriptional regulation in Drosophila. Nature 480: 391*–*395. 10.1038/nature10492 22056986 PMC4082306

[bib7] Chow J. C. Ciaudo C. Fazzari M. J. Mise N. Servant N., 2010 LINE-1 activity in facultative heterochromatin formation during X chromosome inactivation. Cell 141: 956–969. 10.1016/j.cell.2010.04.042 20550932

[bib8] Deng H. Zhang W. Bao X. Martin J. N. Girton J., 2005 The JIL-1 kinase regulates the structure of *Drosophila* polytene chromosomes. Chromosoma 114: 173–182. 10.1007/s00412-005-0006-8 15986206

[bib9] Deng X. Meller V. H., 2006 roX RNAs are required for increased expression of X-linked genes in *Drosophila melanogaster* males. Genetics 174: 1859–1866. 10.1534/genetics.106.064568 17028315 PMC1698640

[bib10] Deng X. Rattner B. P. Souter S. Meller V. H., 2005 The severity of roX1 mutations is predicted by MSL localization on the X chromosome. Mech. Dev. 122: 1094–1105. 10.1016/j.mod.2005.06.004 16125915

[bib11] Deuring R. Fanti L. Armstrong J. A. Sarte M. Papoulas O., 2000 The ISWI chromatin-remodeling protein is required for gene expression and the maintenance of higher order chromatin structure in vivo. Mol. Cell 5: 355–365. 10.1016/S1097-2765(00)80430-X 10882076

[bib12] de Wit E. Greil F. van Steensel B., 2005 Genome-wide HP1 binding in *Drosophila*: developmental plasticity and genomic targeting signals. Genome Res. 15: 1265–1273. 10.1101/gr.3198905 16109969 PMC1199541

[bib13] DiBartolomeis S. M. Tartof K. D. Jackson F. R., 1992 A superfamily of *Drosophila* satellite related (SR) DNA repeats restricted to the X chromosome euchromatin. Nucleic Acids Res. 20: 1113–1116. 10.1093/nar/20.5.1113 1549474 PMC312099

[bib14] Ebert A. Schotta G. Lein S. Kubicek S. Krauss V., 2004 Su(var) genes regulate the balance between euchromatin and heterochromatin in *Drosophila*. Genes Dev. 18: 2973–2983. 10.1101/gad.323004 15574598 PMC534657

[bib15] Figueiredo M. L. Kim M. Philip P. Allgardsson A. Stenberg P., 2014 Non-coding roX RNAs prevent the binding of the MSL-complex to heterochromatic regions. PLoS Genet. 10: e1004865 10.1371/journal.pgen.1004865 25501352 PMC4263465

[bib16] Gallach M., 2014 Recurrent turnover of chromosome-specific satellites in *Drosophila*. Genome Biol. Evol. 6: 1279–1286. 10.1093/gbe/evu104 24846631 PMC4079201

[bib17] Gelbart M. E. Kuroda M. I., 2009 *Drosophila* dosage compensation: a complex voyage to the X chromosome. Development 136: 1399–1410. 10.1242/dev.029645 19363150 PMC2674252

[bib18] Hsieh T. Brutlag D., 1979 Sequence and sequence variation within the 1.688 g/cm3 satellite DNA of *Drosophila melanogaster*. J. Mol. Biol. 135: 465–481. 10.1016/0022-2836(79)90447-9 231676

[bib19] Jin Y. Wang Y. Johansen J. Johansen K. M., 2000 JIL-1, a chromosomal kinase implicated in regulation of chromatin structure, associates with the male specific lethal (MSL) dosage compensation complex. J. Cell Biol. 149: 1005–1010. 10.1083/jcb.149.5.1005 10831604 PMC2174831

[bib20] Joshi S. S. Meller V. H., 2017 Satellite repeats identify X chromatin for dosage compensation in *Drosophila melanogaster* males. Curr. Biol. 27: 1393–1402.e2. 10.1016/j.cub.2017.03.078 28457869 PMC5497753

[bib21] Kelley R. L. Kuroda M. I., 2003 The *Drosophila* roX1 RNA gene can overcome silent chromatin by recruiting the male-specific lethal dosage compensation complex. Genetics 164: 565–574. 12807777 10.1093/genetics/164.2.565 PMC1462573

[bib22] Kelley R. L. Meller V. H. Gordadze P. R. Roman G. Davis R. L., 1999 Epigenetic spreading of the *Drosophila* dosage compensation complex from roX RNA genes into flanking chromatin. Cell 98: 513–522. 10.1016/S0092-8674(00)81979-0 10481915

[bib23] Kind J. Akhtar A., 2007 Cotranscriptional recruitment of the dosage compensation complex to X-linked target genes. Genes Dev. 21: 2030–2040. 10.1101/gad.430807 17699750 PMC1948858

[bib24] Koya S. K. Meller V. H., 2011 roX RNAs and genome regulation in *Drosophila melanogaster*. Prog. Mol. Subcell. Biol. 51: 147–160. 10.1007/978-3-642-16502-3_7 21287137

[bib25] Koya S. K. Meller V. H., 2015 Modulation of heterochromatin by male specific lethal proteins and roX RNA in *Drosophila melanogaster* males. PLoS One 10: e0140259 10.1371/journal.pone.0140259 26468879 PMC4607463

[bib26] Kuhn G. C. Kuttler H. Moreira-Filho O. Heslop-Harrison J. S., 2012 The 1.688 repetitive DNA of *Drosophila*: concerted evolution at different genomic scales and association with genes. Mol. Biol. Evol. 29: 7–11. 10.1093/molbev/msr173 21712468

[bib27] Kuzu G. Kaye E. G. Chery J. Siggers T. Yang L., 2016 Expansion of GA dinucleotide repeats increases the density of CLAMP binding sites on the X–chromosome to promote *Drosophila* dosage compensation. PLoS Genet. 12: e1006120 10.1371/journal.pgen.1006120 27414415 PMC4945028

[bib28] Larschan E. Alekseyenko A. A. Gortchakov A. A. Peng S. Li B., 2007 MSL complex is attracted to genes marked by H3K36 trimethylation using a sequence-independent mechanism. Mol. Cell 28: 121–133. 10.1016/j.molcel.2007.08.011 17936709

[bib29] Larschan E. Bishop E. P. Kharchenko P. V. Core L. J. Lis J. T., 2011 X chromosome dosage compensation via enhanced transcriptional elongation in *Drosophila*. Nature 471: 115–118. 10.1038/nature09757 21368835 PMC3076316

[bib30] Lee J. T., 2009 Lessons from X-chromosome inactivation: long ncRNA as guides and tethers to the epigenome. Genes Dev. 23: 1831–1842. 10.1101/gad.1811209 19684108 PMC2725936

[bib31] Lim C. K. Kelley R. L., 2013 The *Drosophila* over compensating males gene genetically inhibits dosage compensation in males. PLoS One 8: e60450 10.1371/journal.pone.0060450 23565249 PMC3615101

[bib32] Liu L. P. Ni J. Q. Shi Y. D. Oakeley E. J. Sun F. L., 2005 Sex-specific role of *Drosophila melanogaster* HP1 in regulating chromatin structure and gene transcription. Nat. Genet. 37: 1361–1366. 10.1038/ng1662 16258543

[bib33] Lucchesi J. C. Kuroda M. I., 2015 Dosage compensation in Drosophila. Cold Spring Harb. Perspect. Biol. 7: a019398 10.1101/cshperspect.a019398 25934013 PMC4448616

[bib34] Lucchesi J. C. Kelly W. G. Panning B., 2005 Chromatin remodeling in dosage compensation. Annu. Rev. Genet. 39: 615–651. 10.1146/annurev.genet.39.073003.094210 16285873

[bib35] Lyon M. F., 1998 X-chromosome inactivation: a repeat hypothesis. Cytogenet. Cell Genet. 80: 133–137. 10.1159/000014969 9678347

[bib36] McElroy K. A. Jung Y. L. Zee B. M. Wang C. I. Park P. J., 2017 upSET, the *Drosophila* homologue of SET3, is required for viability and the proper balance of active and repressive chromatin marks. G3 (Bethesda) 7: 625–635. 10.1534/g3.116.037788 28064188 PMC5295607

[bib37] Meller V. H., 2003 Initiation of dosage compensation in *Drosophila* embryos depends on expression of the roX RNAs. Mech. Dev. 120: 759–767. 10.1016/S0925-4773(03)00157-6 12915227

[bib38] Meller V. H. Rattner B. P., 2002 The roX genes encode redundant male-specific lethal transcripts required for targeting of the MSL complex. EMBO J. 21: 1084–1091. 10.1093/emboj/21.5.1084 11867536 PMC125901

[bib39] Meller V. H. Joshi S. S. Deshpande N., 2015 Modulation of chromatin by noncoding RNA. Annu. Rev. Genet. 49: 673–695. 10.1146/annurev-genet-112414-055205 26631517

[bib40] Menon D. U. Meller V. H., 2009 Imprinting of the Y chromosome influences dosage compensation in roX1 roX2 *Drosophila melanogaster*. Genetics 183: 811–820. 10.1534/genetics.109.107219 19704014 PMC2778978

[bib41] Menon D. U. Meller V. H., 2012 A role for siRNA in X-chromosome dosage compensation in *Drosophila melanogaster*. Genetics 191: 1023–1028. 10.1534/genetics.112.140236 22554892 PMC3389965

[bib42] Menon D. U. Coarfa C. Xiao W. Gunaratne P. H. Meller V. H., 2014 siRNAs from an X-linked satellite repeat promote X-chromosome recognition in *Drosophila melanogaster*. Proc. Natl. Acad. Sci. USA 111: 16460–16465. 10.1073/pnas.1410534111 25368194 PMC4246271

[bib43] Moazed D., 2009 Small RNAs in transcriptional gene silencing and genome defence. Nature 457: 413–420. 10.1038/nature07756 19158787 PMC3246369

[bib44] Monfort M. Furlong E. E. M. Girardot C., 2017 Dynamix: dynamic visualization by automatic selection of informative tracks from hundreds of genomic datasets. Bioinformatics 33: 2194–2196. 10.1093/bioinformatics/btx141 28334301 PMC5870560

[bib45] Pfaffl M. W., 2001 A new mathematical model for relative quantification in real-time RT-PCR. Nucleic Acids Res. 29: e45 10.1093/nar/29.9.e45 11328886 PMC55695

[bib46] Quinn J. J. Ilik I. A. Qu K. Georgiev P. Chu C., 2014 Revealing long noncoding RNA architecture and functions using domain-specific chromatin isolation by RNA purification. Nat. Biotechnol. 32: 933–940. 10.1038/nbt.2943 24997788 PMC4175979

[bib47] Rastelli L. Richman R. Kuroda M. I., 1995 The dosage compensation regulators MLE, MSL-1 and MSL-2 are interdependent since early embryogenesis in *Drosophila*. Mech. Dev. 53: 223–233. 10.1016/0925-4773(95)00438-7 8562424

[bib48] Seum C. Pauli D. Delattre M. Jaquet Y. Spierer A., 2002 Isolation of Su(var)3–7 mutations by homologous recombination in Drosophila melanogaster. Genetics 161: 1125–1136. 12136016 10.1093/genetics/161.3.1125 PMC1462191

[bib49] Shogren-Knaak M. Ishii H. Sun J. M. Pazin M. J. Davie J. R., 2006 Histone H4–K16 acetylation controls chromatin structure and protein interactions. Science 311: 844–847. 10.1126/science.1124000 16469925

[bib50] Sik Lee Y. Carthew R. W., 2003 Making a better RNAi vector for *Drosophila*: use of intron spacers. Methods 30: 322–329. 10.1016/S1046-2023(03)00051-3 12828946

[bib51] Smith E. R. Pannuti A. Gu W. Steurnagel A. Cook R. G., 2000 The *Drosophila* MSL complex acetylates histone H4 at lysine 16, a chromatin modification linked to dosage compensation. Mol. Cell. Biol. 20: 312–318. 10.1128/MCB.20.1.312-318.2000 10594033 PMC85086

[bib52] Soruco M. M. Chery J. Bishop E. P. Siggers T. Tolstorukov M. Y., 2013 The CLAMP protein links the MSL complex to the X chromosome during *Drosophila* dosage compensation. Genes Dev. 27: 1551–1556. 10.1101/gad.214585.113 23873939 PMC3731544

[bib53] Spierer A. Seum C. Delattre M. Spierer P., 2005 Loss of the modifiers of variegation Su(var)3–7 or HP1 impacts male X polytene chromosome morphology and dosage compensation. J. Cell Sci. 118: 5047–5057. 10.1242/jcs.02623 16234327

[bib54] Spierer A. Begeot F. Spierer P. Delattre M., 2008 SU(VAR)3–7 links heterochromatin and dosage compensation in Drosophila. PLoS Genet. 4: e1000066 10.1371/journal.pgen.1000066 18451980 PMC2320979

[bib55] Stark C. Breitkreutz B. J. Reguly T. Boucher L. Breitkreutz A., 2006 BioGRID: a general repository for interaction datasets. Nucleic Acids Res. 34: D535–D539. 10.1093/nar/gkj109 16381927 PMC1347471

[bib56] Straub T. Grimaud C. Gilfillan G. D. Mitterweger A. Becker P. B., 2008 The chromosomal high-affinity binding sites for the *Drosophila* dosage compensation complex. PLoS Genet. 4: e1000302 10.1371/journal.pgen.1000302 19079572 PMC2586088

[bib57] Sural T. H. Peng S. Li B. Workman J. L. Park P. J., 2008 The MSL3 chromodomain directs a key targeting step for dosage compensation of the *Drosophila melanogaster* X chromosome. Nat. Struct. Mol. Biol. 15: 1318–1325. 10.1038/nsmb.1520 19029895 PMC2636508

[bib58] Swaminathan A. Gajan A. Pile L. A., 2012 Epigenetic regulation of transcription in *Drosophila*. Front. Biosci. 17: 909–937. 10.2741/3964 22201781

[bib59] Verdel A. Jia S. Gerber S. Sugiyama T. Gygi S., 2004 RNAi-mediated targeting of heterochromatin by the RITS complex. Science 303: 672–676. 10.1126/science.1093686 14704433 PMC3244756

[bib60] Wang Y. Zhang W. Jin Y. Johansen J. Johansen K. M., 2001 The JIL-1 tandem kinase mediates histone H3 phosphorylation and is required for maintenance of chromatin structure in *Drosophila*. Cell 105: 433–443. 10.1016/S0092-8674(01)00325-7 11371341

[bib61] Waring G. L. Pollack J. C., 1987 Cloning and characterization of a dispersed, multicopy, X chromosome sequence in *Drosophila melanogaster*. Proc. Natl. Acad. Sci. USA 84: 2843–2847. 10.1073/pnas.84.9.2843 3106978 PMC304756

[bib62] Zhang K. Mosch K. Fischle W. Grewal S. I., 2008 Roles of the Clr4 methyltransferase complex in nucleation, spreading and maintenance of heterochromatin. Nat. Struct. Mol. Biol. 15: 381–388. 10.1038/nsmb.1406 18345014

[bib63] Zhang W. Deng H. Bao X. Lerach S. Girton J., 2006 The JIL-1 histone H3S10 kinase regulates dimethyl H3K9 modifications and heterochromatic spreading in *Drosophila*. Development 133: 229–235. 10.1242/dev.02199 16339185

